# Commentary: The circulatory effects of increased hydrostatic pressure due to immersion and submersion

**DOI:** 10.3389/fphys.2022.1029393

**Published:** 2022-10-18

**Authors:** Robert P. Weenink, Thijs T. Wingelaar

**Affiliations:** ^1^ Diving Medical Center, Royal Netherlands Navy, Den Helder, Netherlands; ^2^ Department of Anesthesiology, Amsterdam University Medical Centers, Amsterdam, Netherlands

**Keywords:** hydrostatic pressure, immersion, blood circulation, hyperbaric oxygenation, swimming, diving, immersion pulmonary edema, rescue collapse

We thank Regnard and others for their thorough appreciation of our article on the circulatory effects of increased hydrostatic pressure due to immersion (Weenink and Wingelaar, 2021) as we are convinced that scientific progress can only be made through the exchange of thoughts (Regnard et al., 2022). It is interesting to note the amount of criticism they subject our statements to. Many of the differences in insight between their group and ours may be reduced to semantics or other trivial factors. We will therefore not counter every detail of the commentary as this would be more suitable for a pro and con manuscript—which we would be more than willing to partake in. Our two groups, however, do seem to have quite opposing views when it comes to the most fundamental issue of our article, i.e., whether hydrostatic pressure is able to exert a compressing force on the vasculature. We strongly believe it cannot and believe the analogy with the hyperbaric chamber perfectly serves to explain this. In our opinion, Regnard et al. too easily dismiss this analogy, thereby disregarding important information that supports our viewpoint.

First and foremost, we would like to stress that in our comparison between wet compression (immersion) and dry compression (in a hyperbaric chamber), we disregard the effects of a vertical pressure gradient. Most certainly, as Regnard et al. point out, this vertical pressure gradient is virtually zero during dry compression, while the pressure increase during vertical immersion in water is 1 cm H_2_O per cm of immersion by definition. However, the point we are making is that hydrostatic pressure, independent of its magnitude or whether it is caused by dry or wet compression, does not selectively squeeze the vascular wall. Pressure is transmitted throughout all tissues (which are indeed of “grossly hydric density”) and does not suddenly stop at the adventitia (which is also of hydric density) (Pascal, 1663). Our analogy between immersion and the hyperbaric chamber serves to demonstrate this. If the small amount of hydrostatic pressure encountered during, for instance, swimming, would be able to compress the vasculature in the dependent body parts, what would happen to the vasculature during deep diving or dry compression to depths far exceeding this pressure (Gardette et al., 1993).

Having said this, we concur with our commentators that changes in fluid distribution and vascular diameters do occur during immersion in a fluid. The commentary aptly points out that the explanation given for these phenomena in our original article was incomplete. We only pointed toward the decreased extravasation of the fluid during immersion as the explanation for increased centralized volume, but clearly, given the time constants, this is not the complete explanation. Although our commentators suggest the decreased vascular diameter in dependent body parts is caused by hydrostatic compression, we believe it is caused by the counteracting of gravity due to buoyancy. When a body part is immersed, gravity naturally still works but is counteracted by buoyancy. The teardrop shape of a water-filled balloon above the surface changes to a sphere when it is submerged (Supplementary Video S1). Gravity normally causes distension of dependent body parts, which is reduced during immersion, centralizing the circulation. No additional explanation such as hydrostatic vascular compression is needed.

Returning to the premise of our original article, it seems that despite our differences, both groups concur that the phenomenon of “rescue collapse” exists and that the removal of a subject from water should be carried out with caution. We seem to be at odds when it comes to the explanation of the phenomenon, which only serves to show the importance of academic debate in this field.

## Supplementary material

Supplementary Video S1 Demonstration of submersion of a water-filled balloon. Above water, the balloon has the shape of a teardrop, which changes into a sphere when the balloon is submerged in water.
